# Author Correction: Human CEACAM1 is targeted by a *Streptococcus pyogenes* adhesin implicated in puerperal sepsis pathogenesis

**DOI:** 10.1038/s41467-023-38372-1

**Published:** 2023-05-09

**Authors:** Erin A. Catton, Daniel A. Bonsor, Carolina Herrera, Margaretha Stålhammar-Carlemalm, Mykola Lyndin, Claire E. Turner, Jo Soden, Jos A. G. van Strijp, Bernhard B. Singer, Nina M. van Sorge, Gunnar Lindahl, Alex J. McCarthy

**Affiliations:** 1 Centre for Bacterial Resistance Biology, Section of Molecular Microbiology, Department of Infectious Diseases, Imperial College London, London, SW7 2AZ UK; 2 University of Maryland, Baltimore, MD 21201 USA; 3 NCI RAS Initiative, Cancer Research Technology Program, Frederick National Laboratory for Cancer Research, Frederick, MD USA; 4 Section of Immunology of Infection, Department of Infectious Disease, Imperial College London, London, W2 1NY UK; 5 Department of Laboratory Medicine, Division of Medical Microbiology, Lund University, Lund, 223 62 Sweden; 6 Sumy State University, Sumy, 40000 Ukraine; 7 Institute of Anatomy, Medical Faculty, University of Duisburg-Essen, Essen, 45147 Germany; 8 The School of Biosciences, The Florey Institute, The University of Sheffield, Sheffield, S10 2TN UK; 9 Retrogenix, Chinley, High Peak, SK23 6FJ Chinley, UK; 10 Department of Medical Microbiology, UMC Utrecht, Utrecht, 3584 CX The Netherlands; 11 Department of Medical Microbiology and Infection Prevention, Amsterdam UMC location University of Amsterdam, Amsterdam Institute for Infection and Immunity, Amsterdam, 1105 AZ The Netherlands; 12 Netherlands Reference Laboratory for Bacterial Meningitis, Amsterdam UMC, location AMC, Amsterdam, 1105 AZ The Netherlands; 13 Department of Chemistry, Division of Applied Microbiology, Lund University, Lund, 221 00 Sweden

**Keywords:** Bacterial pathogenesis, Infection, Pathogens, Bacterial infection

Correction to: *Nature Communications* 10.1038/s41467-023-37732-1, published online 20 April 2023

In this article the affiliation details for Bernhard B. Singer were incorrectly given as ‘Department of Medical Microbiology and Infection Prevention, Amsterdam UMC location University of Amsterdam, Amsterdam Institute for Infection and Immunity, Amsterdam, 1105 AZ, The Netherlands’ but should have been ‘Institute of Anatomy, Medical Faculty, University of Duisburg-Essen, Essen, 45147, Germany’.

The original article has been corrected.

