# Supplementary material for: Human CEACAM1 is targeted by a Streptococcus pyogenes adhesin implicated in puerperal sepsis pathogenesis
Source: Nat Commun. 2023 Apr 20;14:2275. doi: 10.1038/s41467-023-37732-1 (PMC10119177; doi:10.1038/s41467-023-37732-1)
Supplement: Supplementary file 1 — Supplementary Information [file 41467_2023_37732_MOESM1_ESM.pdf]

Supplemental Material

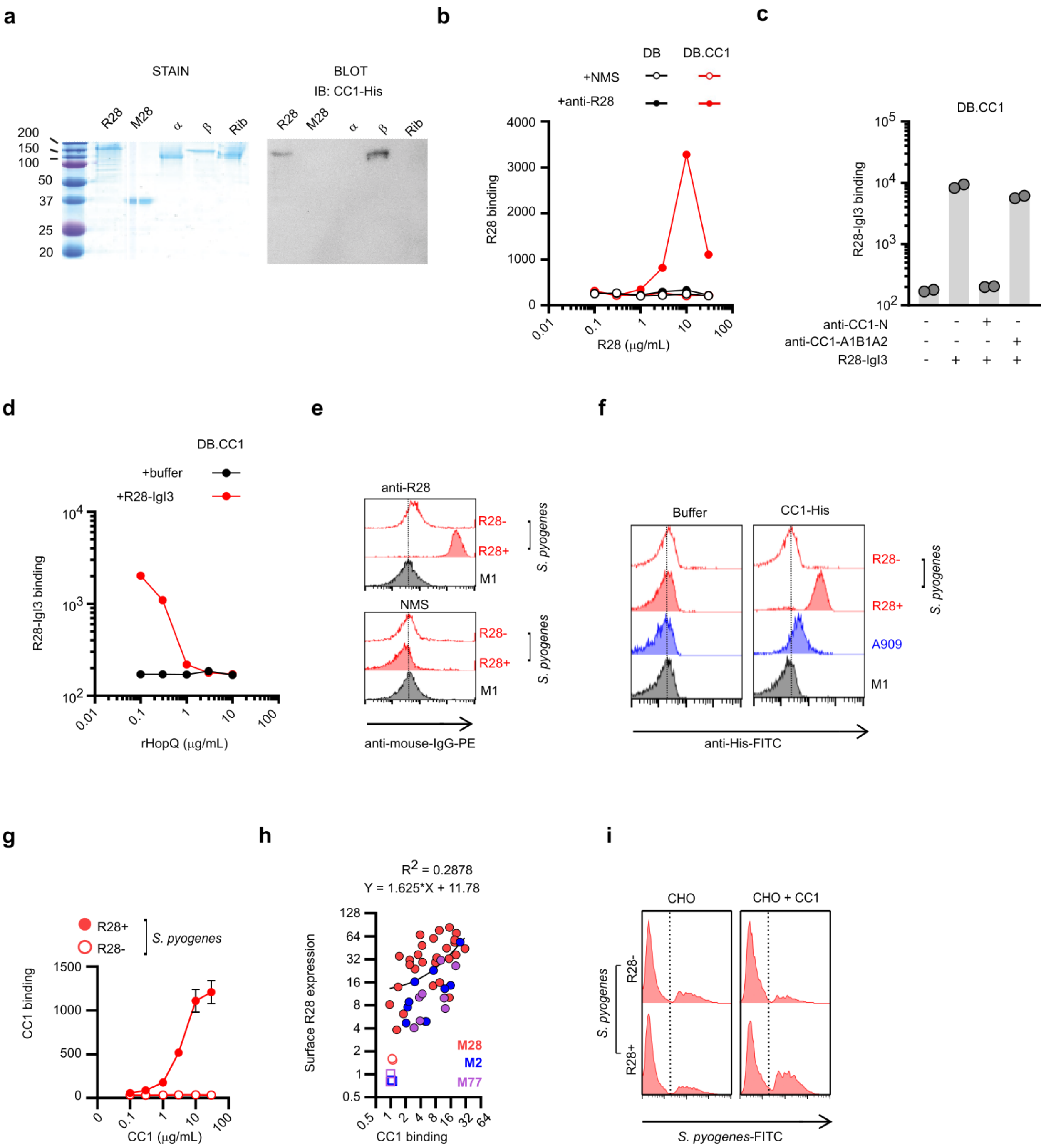

Supplementary Fig. 1. R28 binds CEACAM1.

a Western blot analysis was performed on purified streptococcal proteins R28, M28, β, α and Rib using the rCEACAM1 (CC1) as probe. The β protein of *S. agalactiae* was used as positive control. Representative of 2 independent experiments. **b** Binding of purified R28 to dynabeads (DB) coated with rCC1 (DB.CC1) or control (DB) quantified by flow cytometric analysis. Mean values

from *n* = 2 independent replicates. **c** **(**Pre-) incubation of DB.CC1 with a monoclonal antibody (mAb) specific to the N-terminal domain of CC1, CC3 and CC5 prevents the binding of R28-IgI3. Data are represented as mean ± s.d. of *n* = 2 independent replicates. **d** (Pre-)incubation of DB.CC1 with rHopQ, a protein from *H. pylori*, which binds CC1-N, blocks the binding of R28-IgI3. Mean of *n* = 2 independent replicates. **e** Expression of surface-localized R28 on isogenic *S. pyogenes* AL368 strains quantified by flow cytometric analysis. Representative flow cytometry plots are shown. **f** Binding of rCC1-HIS to a panel of streptococcal strains quantified using flow cytometry analysis. Strains used are *S. pyogenes* AL368 (*S. pyogenes*; M28, R28+) and its Δ*spr28* variant (Δ*spr28;* M28, R28-), *S. pyogenes* M1 (5448, R28-), and *S. agalactiae* A909 (β protein +). Representative flow cytometry plots are shown. **g** Concentration-dependent binding of rCC1-HIS to wildtype *S. pyogenes* AL368 or its Δ*spr28* variant was quantified by flow cytometry. Data are represented as mean ± s.d. from *n* = 3 independent replicates. **h** Correlation between R28 protein expression and rCC1-binding capacity in *S. pyogenes* strains, using values in Fig. 1f and 1g. Closed circles show *spr28+* strains and open circles show isogenic Δ*spr28* strains. Open squares show *spr28-* strains, typed by PCR. **i** Binding of FITC-labelled isogenic wildtype *S. pyogenes* AL368 or its Δ*spr28* variant to CHO or CHO^{CC1} cells at an MOI of 10 quantified by flow cytometry. Representative flow cytometry plots of *n* = 3 replicates are shown.

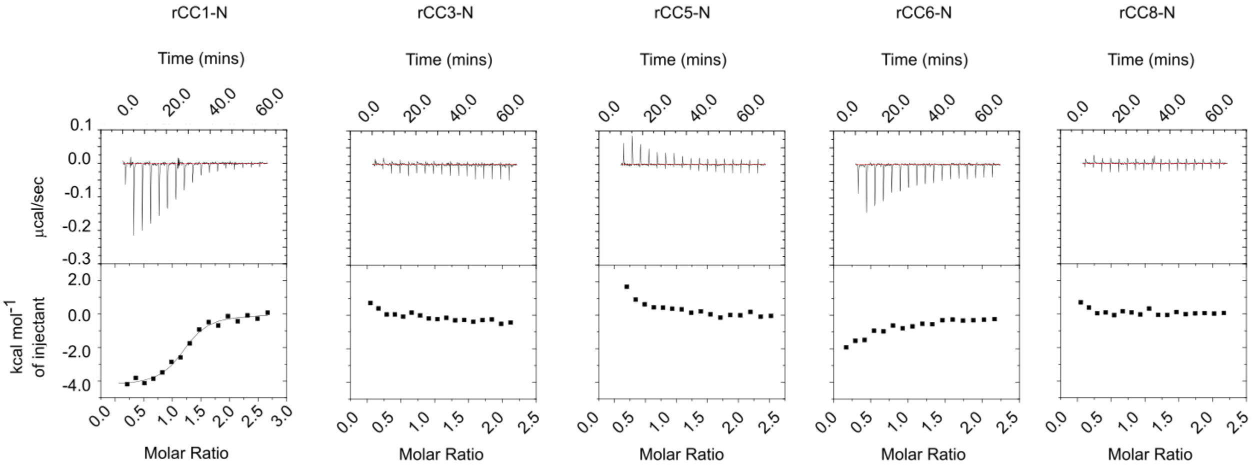

Supplementary Fig. 2. Isothermal calorimetry analysis of R28-IgI3 and CEACAM-N domain interactions. ITC for the titration of rCEACAM1-N (rCC1-N), rCEACAM3-N (rCC3-N), rCEACAM5-N (rCC5-N), rCEACAM6-N (rCC6-N) and rCEACAM8-N (rCC8-N) into R28-IgI3 at 25°C with 16 injections of 2.42 µl aliquots. A thermogram (upper panel) and integrated heats and error bars (middle panel) are shown for each. Our previous ITC assay (left-most panel) revealed that R28-IgI3 and rCC1-N interaction and affinity of $K_D = 1050 \pm 18$ nM, ($\Delta H = -4.8 \pm 0.3$ kcal/mol), and is shown here [17].

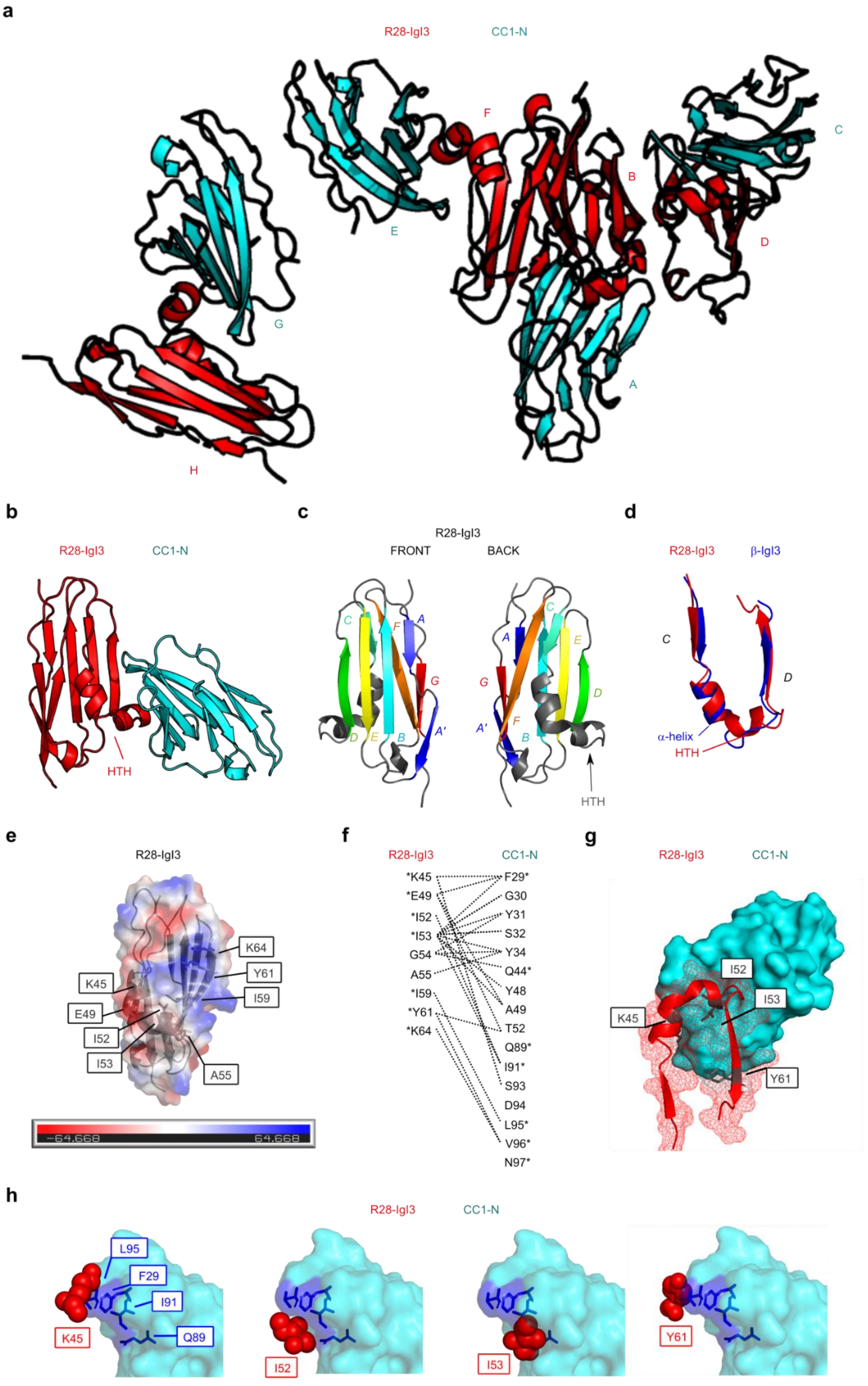
a
R28-IgI3
CC1-N
F
B
C
E
D
G
A
H
b
R28-IgI3
CC1-N
HTH
c
R28-IgI3
FRONT
BACK
C
F
A
G
A'
D
E
B
A
C
E
G
D
A'
F
B
HTH
d
R28-IgI3
β-IgI3
C
D
α-helix
HTH
e
R28-IgI3
K64
Y61
I59
K45
E49
I52
I53
A55
-64.668
64.668
f
R28-IgI3
CC1-N
*K45
*E49
*I52
*I53
G54
A55
*I59
*Y61
*K64
F29*
G30
Y31
S32
Y34
Q44*
Y48
A49
T52
Q89*
I91*
S93
D94
L95*
V96*
N97*
g
R28-IgI3
CC1-N
I52
I53
K45
Y61
h
R28-IgI3
CC1-N
L95
F29
I91
Q89
K45
I52
I53
Y61

Supplementary Fig. 3. Crystal structure of the R28-IgI3 and CEACAM1-N co-complex. **a** Ribbon diagram of the complex. The crystal structure was refined with native data to 3.05 Å resolution, and is formed of four (CC1-N)-(R28-IgI3) complexes. The paired chains are A-B, C-D, E-F and G-H. **b** Crystal structure of R28-IgI3 and the N-terminal domain of CEACAM1 (CC1) shown in ribbon format and showing the helix-turn-helix (HTH). **c** Molecular structure of R28-IgI3, in which each β-strand is colored differently. The HTH (shown in grey) is located between the *C* strand and the *D* strand. **d** Superposition of the *C* to *D* loop of R28-IgI3 and β-IgI3. Critical residues in R28-IgI3 and β-IgI3 required for binding to CC1-N are highlighted. **e** Charged surface electrostatic potential representation of R28-IgI3, in which red is negatively charged, blue is positively charged and white is no charge. The ribbon structure of R28-IgI3 is shown in grey below the surface. Residues identified as important for CC1-N binding are shown. **f** van der Waals forces (dashed lines) between R28-IgI3 and CC1-N residues within 4Å are shown. Residues with * were selected for mutation analysis, and included additional CC1-N mutants available. **g** Surface structure of CC1-N is shown in cyan, and surface mesh structure of R28-IgI3 is shown in red. Key residues in R28-IgI3 required for binding (K45, I52, I53 and Y61) are indicated. **h** Detailed representations focusing on each of the critical R28-IgI3 residues K45, I52, I53 and Y61 and interacting residues in CC1-N (F29, Q89, I91 and L95).

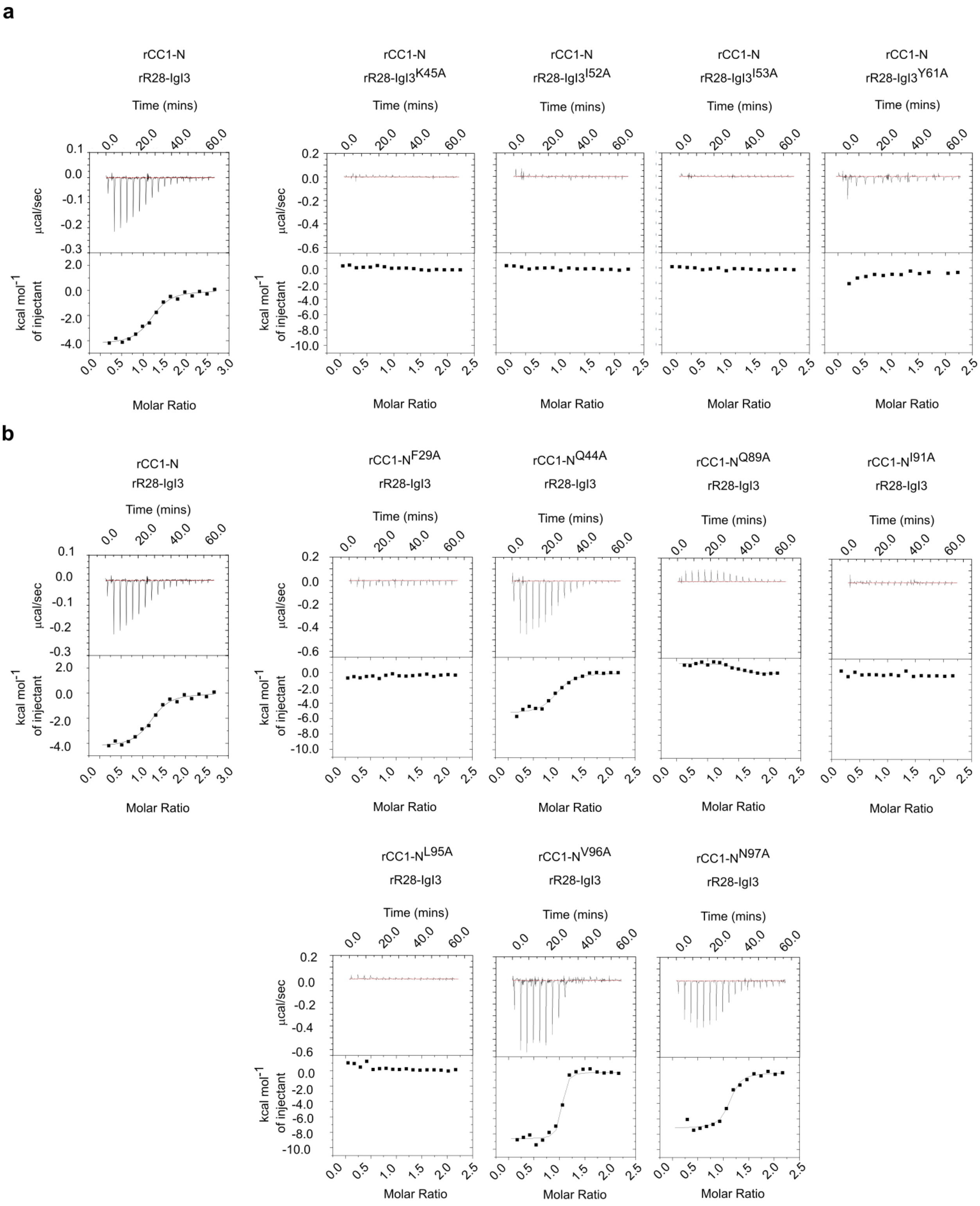

Supplementary Fig. 4. Isothermal calorimetry analysis of the R28-IgI3 and CEACAM-N domain interface.

a ITC for the titration of rCEACAM1-N (rCC1-N) wildtype and variants into R28-IgI3 at 25°C with 16 injections of 2.42 µl aliquots. A thermogram (upper panel) and integrated heats and error bars (middle panel) are shown for each. **b** ITC for the titration of rR28-IgI3 wildtype and variants into rCC1-N at 25°C with 16 injections of 2.42 µl aliquots. A thermogram (upper panel) and integrated heats and error bars (middle panel) are shown for each. Our previous ITC assay revealed that R28-IgI3 and rCC1-N interaction and affinity of $K_D = 1050 \pm 18$ nM, ($\Delta H = -4.8 \pm 0.3$ kcal/mol), and is shown in a and b [17].

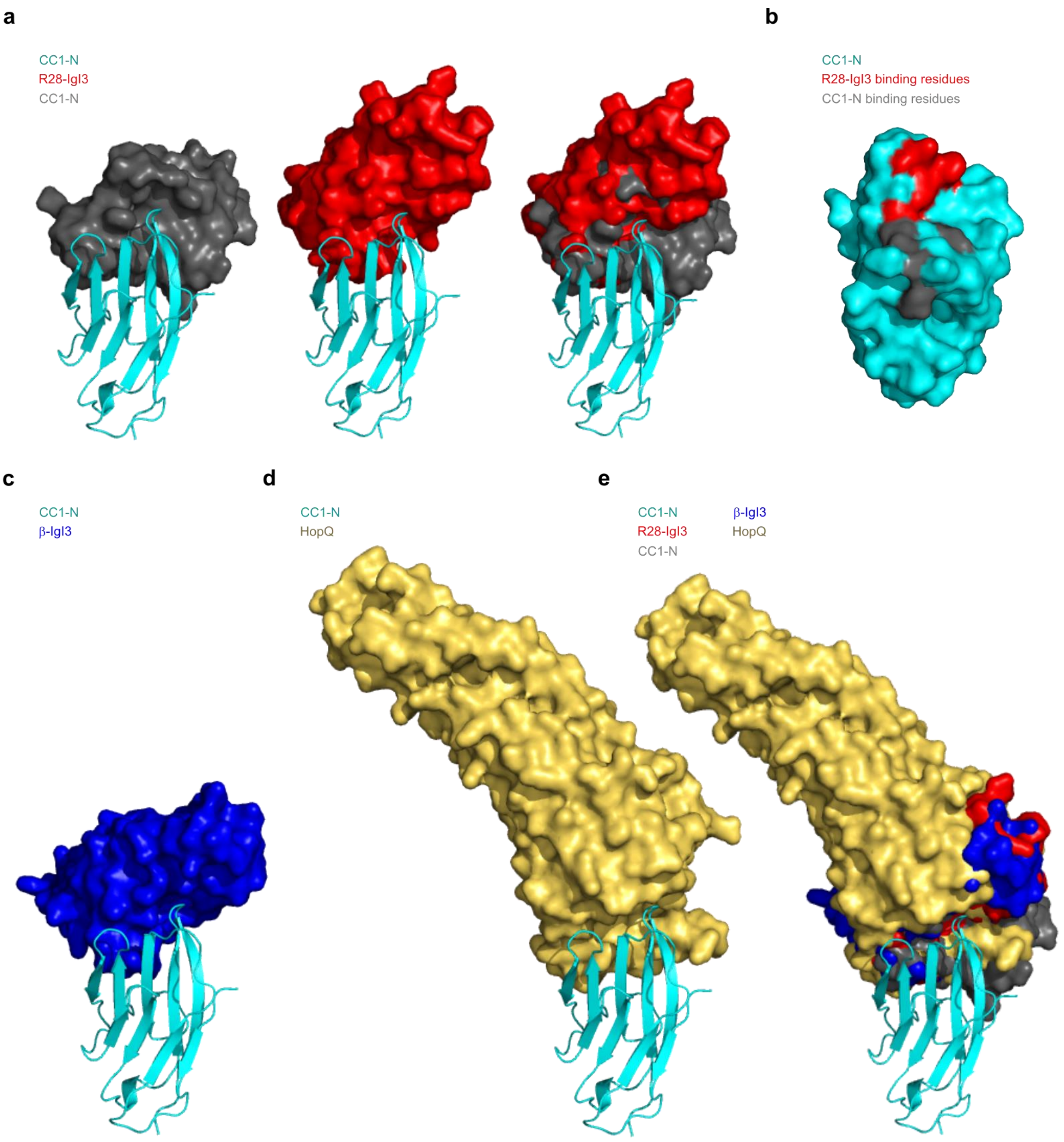

Supplementary Fig. 5. R28-IgI3 binds the *trans* dimerization interface of CEACAM1.

a Superimposition of the crystallographic trans CEACAM1-N (CC1-N) dimer (PDB: 4WHD [https://www.rcsb.org/structure/4WHD]) with the (R28-IgI3)-(CC1-N) structure. **b** Surface structure of CC1-N showing critical residues for forming the *trans* CC1-N dimer or R28-IgI3 interaction. **c** Crystal structure of CC1-N domain in complex with β-IgI3 (PDB: 6V3P

[https://www.rcsb.org/structure/6V3P]). **d** Crystal structure of CC1-N domain in complex with HopQ (PDB: 6AW2 [https://www.rcsb.org/structure/6AW2]). **e** Superimposition of the crystallographic *trans* CEACAM1-N (CC1-N) dimer with the (R28-IgI3)-(CC1-N) structure, (β-IgI3)-(CC1-N) and (HopQ)-(CC1-N) structure.

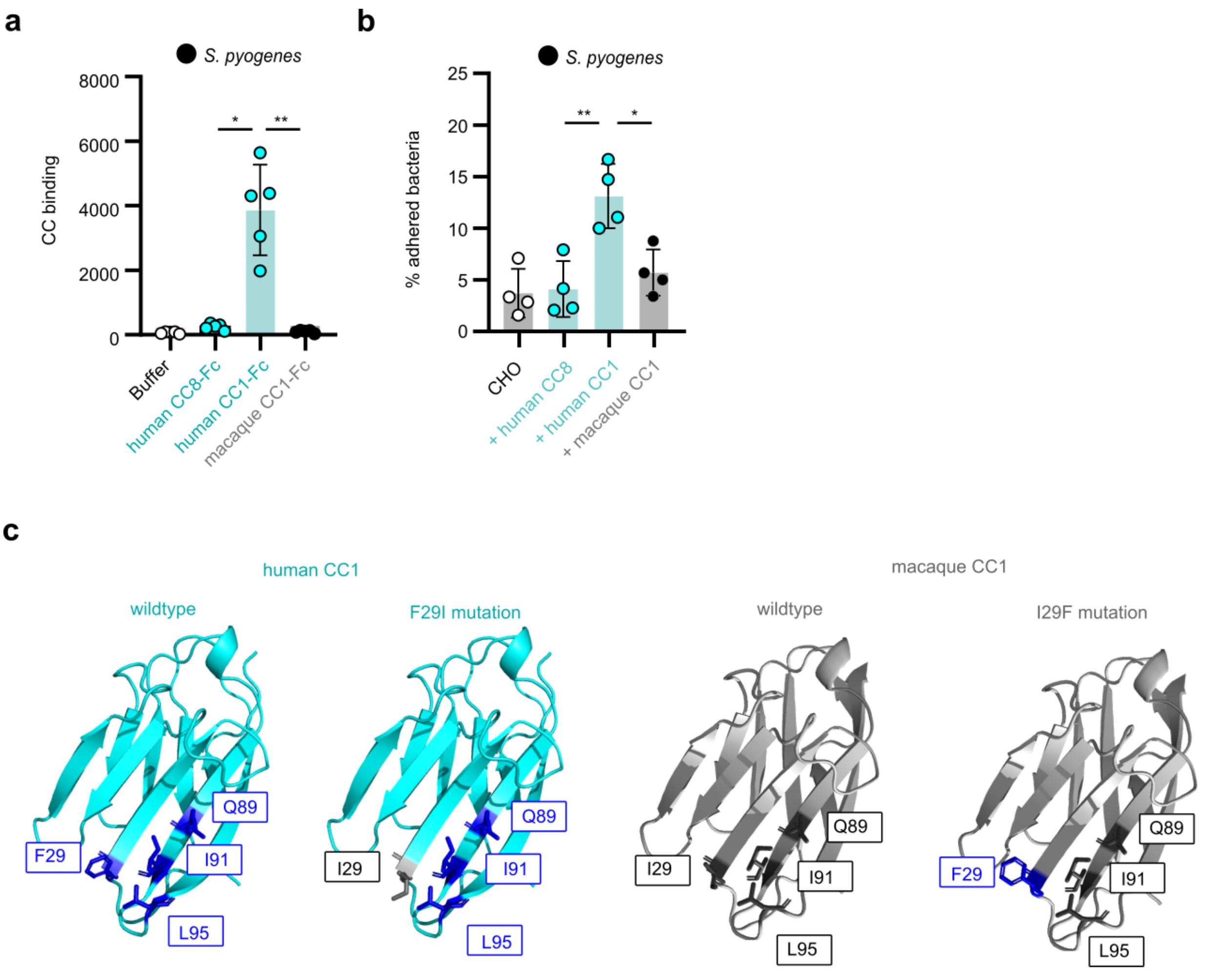

Supplementary Fig. 6. R28-IgI3 binds to human but not macaque CEACAM1.

a Binding of Fc-tagged human and macaque rCC1 to *S. pyogenes* AL368 strain quantified by flow cytometry analysis. Mean ± s.d. of $n = 5$ independent experiments. Statistical significance tested by one-way ANOVA with Tukey's *post-hoc* test human CC1 vs. human CC8 $*p = 0.0117$, human CC1 vs. macaque CC1 $** \ p = 0.0091$). **b** Adherence of wildtype *S. pyogenes* AL368 to CHO, $CHO^{Human\text{-}CC1}$, $CHO^{Human\text{-}CC8}$ and $CHO^{Macaque\text{-}CC1}$ cells at a multiplicity of infection (MOI) of 10 was quantified as the percentage of inoculum (Mean ± s.d. from $n = 4$ independent experiments). Statistical significance tested by one-way ANOVA with Tukey's *post-hoc* test (human CC1 vs. human CC8 $**p = 0.0019$, human CC1 vs. macaque CC1 $* \ p = 0.0215$). **c** Structures of wildtype human CEACAM1-N (CC1-N) and its F29I variant, and of wildtype macaque CC1-N and its I29F

variant. Residues in human CC1-N critical for R28-IgI3 binding, and the corresponding residues in macaque CC1-N, are shown in stick format.

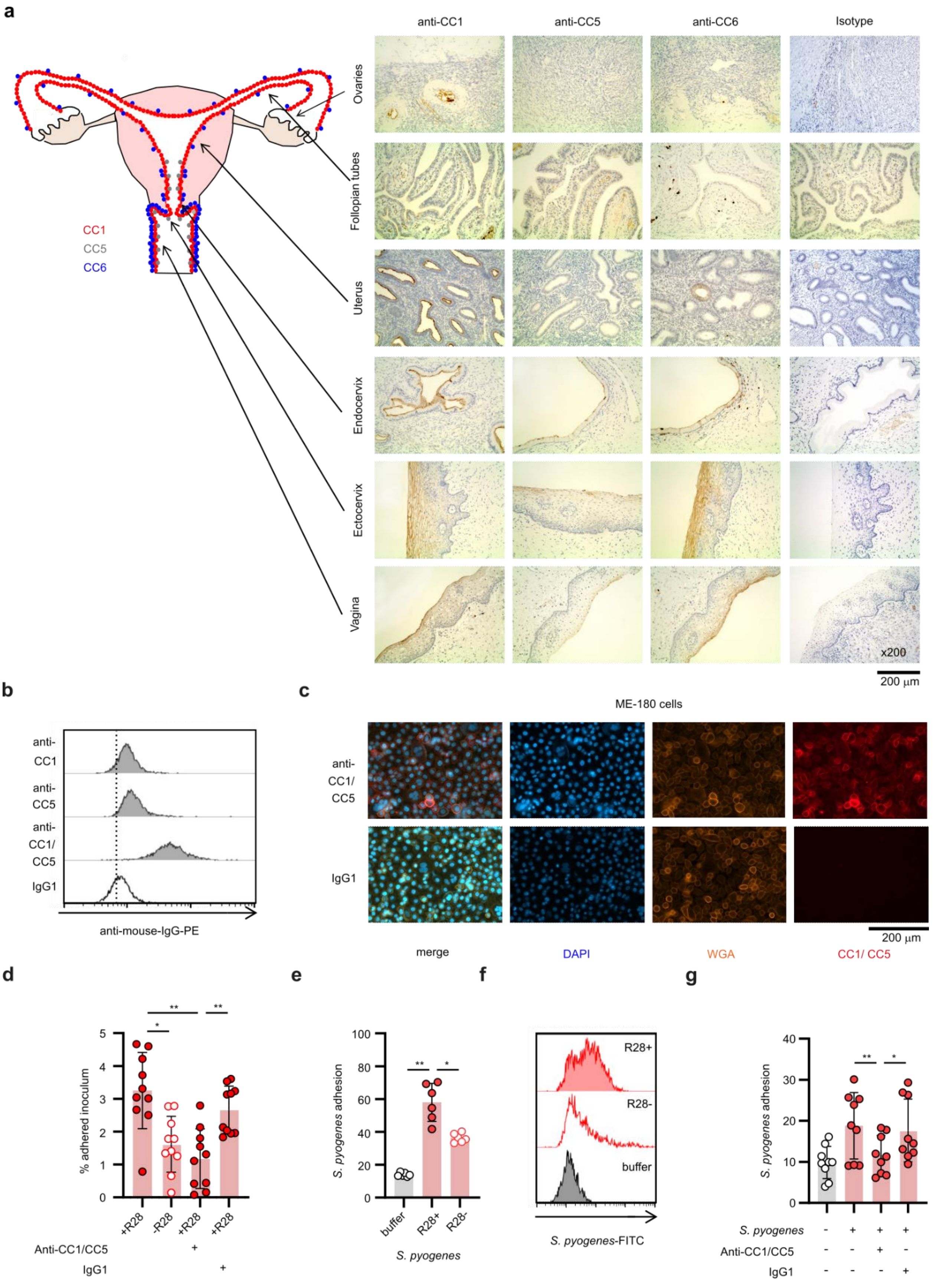
a
anti-CC1
anti-CC5
anti-CC6
Isotype
Ovaries
Follopian tubes
Uterus
Endocervix
Ectocervix
Vagina
CC1
CC5
CC6
x200
200 μm
b
anti-CC1
anti-CC5
anti-CC1/CC5
IgG1
anti-mouse-IgG-PE
c
ME-180 cells
anti-CC1/CC5
IgG1
merge
DAPI
WGA
CC1/ CC5
200 μm
d
% adhered inoculum
+R28
-R28
+R28
+R28
Anti-CC1/CC5
IgG1
e
S. pyogenes adhesion
buffer
R28+
R28-
S. pyogenes
f
R28+
R28-
buffer
S. pyogenes-FITC
g
S. pyogenes adhesion
S. pyogenes
Anti-CC1/CC5
IgG1

Supplementary Fig. 7. R28-CEACAM1 interactions promotes adhesion of *S. pyogenes* to epithelial cells.

a Expression of CEACAM1 (CC1), CEACAM5 (CC5) and CEACAM6 (CC6) in the human female genital tract determined by immunohistochemical examinations using mouse anti- human CC1, CC5, CC6 or isotype control mAb (200X). Representative images from 10 independent experiments. **b** CC1/5 expression on ME-180 cells, assessed by flow cytometry. Representative flow cytometry plots from n = 3 replicates are shown. Expression was measured using antibodies against the N-terminal domains of human CEACAMs. Antibodies utilised are CC1/3/5-Sab of mouse IgG1 type (detects N-terminal domain of human CC1 and CC5), B3-17 of mouse IgG1 type (detects A1B1 domain of CC1), 5C8C4 of mouse IgG1 type (detects unknown region of human CC5) or mouse IgG1 isotype control. **c** CC1/5 expression on ME-180 cells, assessed by fluorescence microscopy. Representative images from 3 independent experiments. **d** Adherence of isogenic *S. pyogenes* 2369-97 strains to ME-180 cells at a multiplicity of infection (MOI) of 10, in the presence or absence a monoclonal antibody (mAb) specific to human CC1 and CC5 N-terminal (Mean ± s.d. of n = 10 independent experiments). Statistical significance tested by one-way ANOVA with Tukey's *post-hoc* test (2369-97 vs. 2369-97Δ*spr28* * p = 0.0291, 2369-97 vs. 2369-97 + CC1/3/5-Sab **p = 0.0023, 2369-97+CC1/3/5-Sab vs. 2369-97 + IgG1 **p = 0.0043). **e and f** Adherence of FITC-labelled wildtype *S. pyogenes* AL368 or its Δ*spr28* variant to detached ME-180 cells at a multiplicity of infection (MOI) of 10 was quantified by flow cytometry analysis. Data in e represent mean ± s.d. from n = 6 independent experiments. Statistical significance tested by one-way ANOVA with Tukey's *post-hoc* test (control vs. R28+ ***p = 0.0004, R28+ vs. R28- *p = 0.0151). Data in f are representative flow cytometry plots. **g** Treatment of ME-180 cells with a mAb specific to the N-terminal domain of CC1 and CC5 impairs binding of FITC-labelled *S. pyogenes* at an MOI of 10 to detached ME-180 cells (Mean ± s.d. of n = 9 independent

experiments). Statistical significance tested by one-way ANOVA with Tukey's *post-hoc* test (R28+ vs. R28+ anti-CC1/CC5 *$p = 0.0139$, R28+ anti-CC1/CC5 vs. R28+ IgG1 **$p = 0.0029$.)

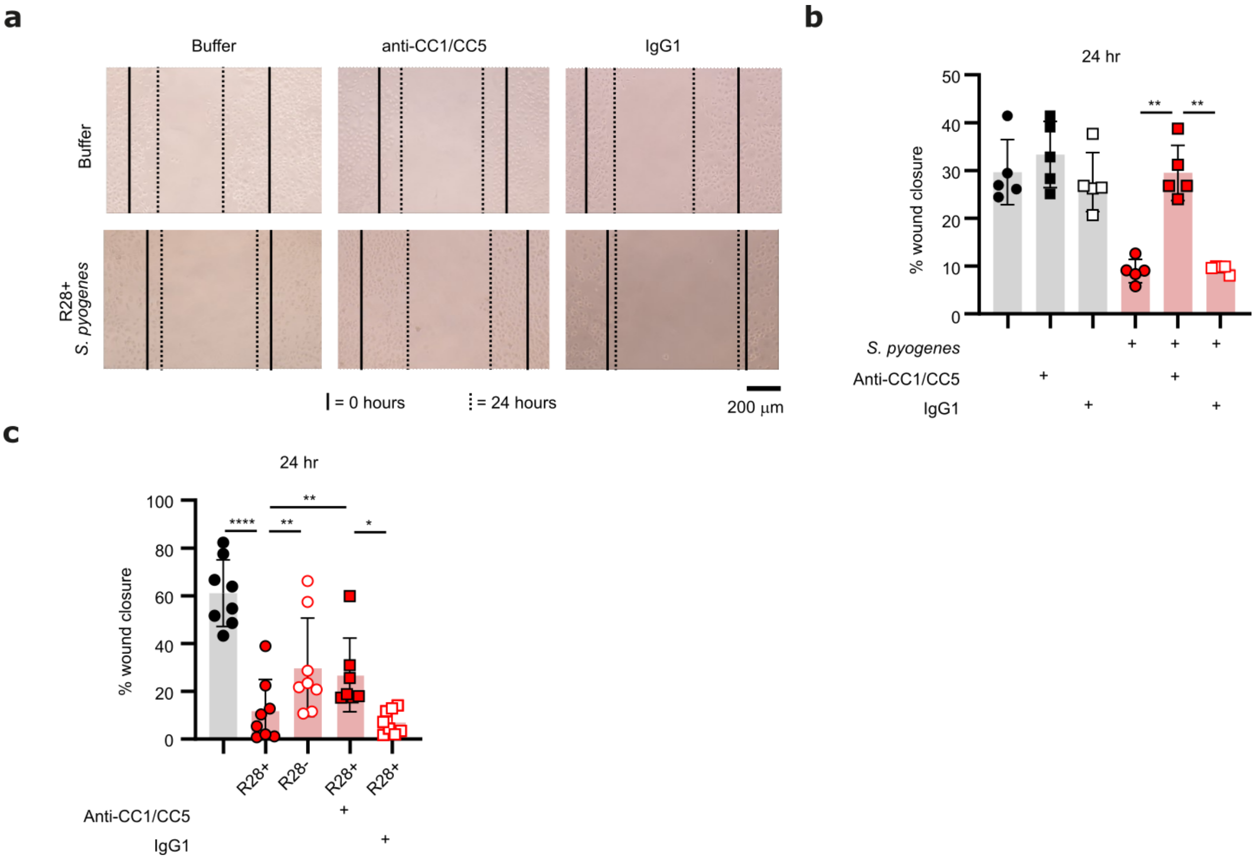

Supplementary Fig. 8. Delayed wound repair requires CEACAM1 accessibility.

a and **b** Treatment of ME-180 cells with a mAb specific to the N-terminal domain of human CC1 and CC5 suppresses the ability of *S. pyogenes* strain AL368 to delay wound repair. A shows representative light microscopy images of ME-180 epithelia at 10X magnification, in which the front of each scratch at 0 hours is marked by a complete line and at 24 or 48 hours by a dashed line, are shown. b shows the mean ± s.d. of $n = 5$ independent experiments. Statistical significance tested by one-way ANOVA with Tukey's *post-hoc* test (*S. pyogenes* vs. *S. pyogenes* + anti-CC1/5-Sab $^{**}p = 0.0024$, *S. pyogenes* + anti-CC1/5-Sab vs. *S. pyogenes* + IgG1 $^{**}p = 0.0096$). **c** Wound healing of ME-180 cell monolayers upon challenge with isogenic *S. pyogenes* 2369-97 strains. ME-180 cells were pre-treated with a mAb specific to the N-terminal domain of human CC1 and CC5, or isotype control, as shown (Mean ± s.d. of $n = 8$ independent experiments). Statistical

significance tested by one-way ANOVA with Tukey's *post-hoc* test (Control vs. R28+ **** p = 0.0001, R28+ vs. R28- **p = 0.00196, R28+ vs. R28+ anti-CC1/5 **p = 0.002, R28+ anti-CC1/5 vs. R28+ IgG1 *p = 0.0297).

a

b

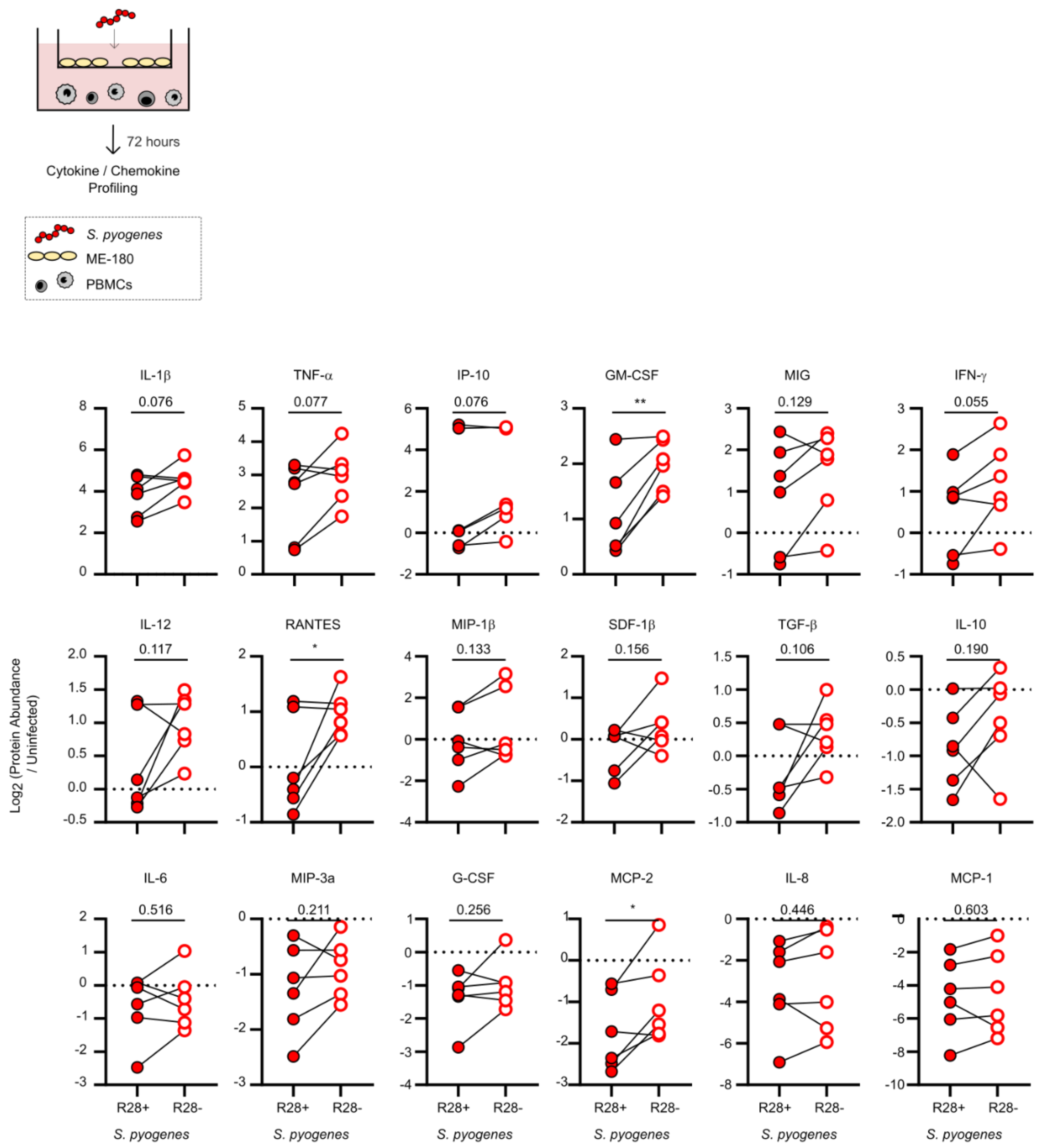

Supplementary Fig. 9. Transwell assay measuring cytokine/chemokine responses to *S. pyogenes*

a Schematic representation of measurement of the cytokine / chemokine response of wounded ME-180 cell monolayers (cultured on transwell inserts) and human PBMCs (basal compartment) upon challenge with γ-irradiated *S. pyogenes* AL368 strains or control buffer. **b** Quantification of

the cytokine / chemokine protein level production by wounded human cervical epithelial cell monolayers and primary human PBMCs in response to infection with γ-irradiated *S. pyogenes* strains. Samples were normalized against uninfected controls. Data are mean and standard deviation from $n = 3$ independent human PBMC donors. Statistical analysis by paired two-tailed Student *t* test, (GM-CSF $^{**}p = 0.0063$, RANTES $^{*}p = 0.0439$, MCP-2 $^{*}p = 0.0329$).

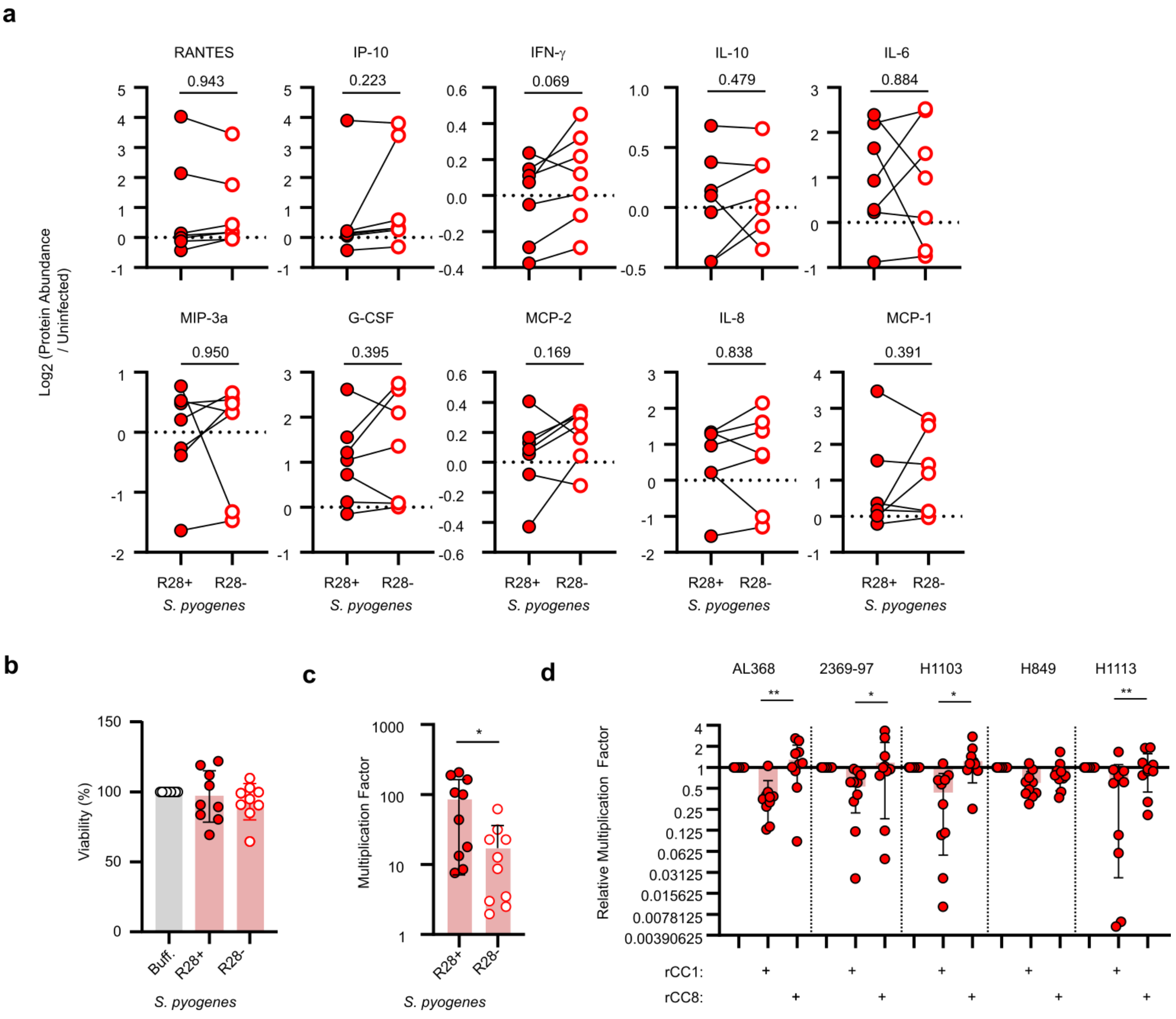

Supplementary Fig. 10. ***Ex vivo*** **cervical explant cytokine/chemokine responses to** ***S. pyogenes***

a Quantification of the cytokine / chemokine protein level production by wounded human ecto-cervical explants in response to infection with *S. pyogenes* AL368 strains. Samples were normalized against uninfected controls. Data are mean and s.d. from n = 7 independent human donors. Statistical analysis by paired two-tailed Student t test, where p values are shown. **b** Effects of *S. pyogenes* infections on human ecto-cervical explant viability as determined using the MTT assay. After incubation at shown time periods, the effect of *S. pyogenes* on tissue viability was determined by comparing the viability of the treated explants to that of the untreated tissue control (same donor). Data are mean and ± s.d. of 2 technical replicates from n = 5 independent human

donors. **c** Replication of isogenic *S. pyogenes* 2369-97 strains in human whole blood was quantified after 3 hours as the percentage of inoculum (Mean ± s.d. from $n = 10$ independent experiments). Statistical significance tested by paired two-tailed Student's *t* test (* $p = 0.0288$). **d** Replication of *S. pyogenes* strains in human whole blood after being pre-incubated with or without rCC1 or rCC8, was quantified after 3 hours as the percentage of inoculum. Data was normalized against untreated control (Mean ± s.d. from $n = 10$ independent experiments). Statistical significance tested by paired two-tailed Student's *t* test (AL368 **$p = 0.0043$, 2369-97 *$p = 0.043$, H1103 *$p = 0.0114$, H849 **$p = 0.0038$).

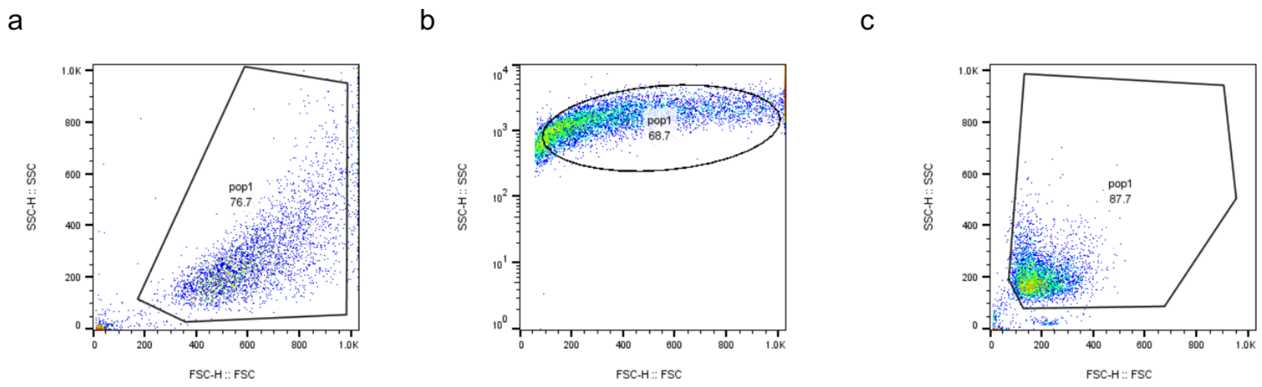

Supplementary Fig. 11. Representative gating stratergies. a ME-180 cells / CHO cells. **b** bacteria. **c** neutrophils.

Supplementary Table 1. Data collection and refinement statistics for R28-IgI3 and CEACAM1 (CC1)-N. Values in parentheses are for highest-resolution shell.

	**R28-IgI3 and CEACAM1-N**
**Data collection**	
Space group	P43
Cell dimensions	
a, b, c (Å)	76.96, 76.96, 334.85
α, β, γ (°)	90, 90, 90
Resolution (Å)	38.96-3.05 (3.19-3.05)
R_{merge}	0.076 (0.909)
$R_{p.i.m}$	0.051 (0.623)
$I / \sigma I$	16.0 (1.9)
Completeness (%)	99.9 (99.7)
Redundancy	6.2 (6.1)
$CC_{1/2}$	0.999 (0.828)
**Refinement**	
Resolution (Å)	38.96-3.05 (3.19-3.05)
No. reflections	36875
R_{work} / R_{free}	20.7/23.8
No. atoms	
Protein	6614
Water	9
Ligand	103
B-factors	
Protein	101.8
Water	79.1
Ligands	115.5
R.m.s. deviations	
Bond lengths (Å)	0.002
Bond angles (°)	0.529

Supplementary Table 2. RMSD values of two chain complexes.

	**A-C**	**B-D**	**E-F**	**G-H**
**A-C**	-	0.190 Å	0.403 Å	0.321 Å
**B-D**	-	-	0.406 Å	0.397 Å
**E-F**	-	-	-	0.188 Å
**G-H**	-	-	-	-

Supplementary Table 3: Isothermal Titration Calorimetry (ITC) binding curves constants and thermodynamic paramters for R28-IgI3 and CEACAM1 (CC1)-N mutants. Experiments were performed using an iTC200 instrument (GE Healthcare), at 25 °C with 16 injections of 2.42 µL aliquots. All data were analyzed using Origin 7.0 software.

	K_D (nM)	ΔH (kcal mol^{-1})	TΔS (kcal mol^{-1})
**Binding to CC1-N**			
R28-IgI3	1050±18	-4.8±0.4	+3.4
R28-IgI3^{K45A}		No Binding Observed	
R28-IgI3^{I52A}		No Binding Observed	
R28-IgI3^{I53A}		No Binding Observed	
R28-IgI3^{Y61A}		No Binding Observed	
**Binding to β-IgI3**			
rCC1-N	1050±18	-4.8±0.4	+3.4
rCC1-N^{F29A}		No Binding Observed	
rCC1-N^{Q44A}	920±400	-6.3±0.9	+1.8
rCC1-N^{Q89A}	687±270	+1.4±0.1	+9.8
rCC1-N^{I91A}		No Binding Observed	
rCC1-N^{L95A}		No Binding Observed	
rCC1-N^{V96A}	116±7	-8.5±0.1	+1.0
rCC1-N^{N97A}	210±68	-7.4±0.2	+1.7
