# Supplementary material for: Human CEACAM1 is targeted by a Streptococcus pyogenes adhesin implicated in puerperal sepsis pathogenesis
Source: Nat Commun. 2023 Apr 20;14:2275. doi: 10.1038/s41467-023-37732-1 (PMC10119177; doi:10.1038/s41467-023-37732-1)
Supplement: Supplementary file 3 — Description of Additional Supplementary Files [file 41467_2023_37732_MOESM3_ESM.pdf]

**Description of Additional Supplementary Files**

File Name: Supplementary Data 1
Description: Human membrane proteins screened. 3359 clones from 2625 genes.

File Name: Supplementary Data 2
Description: Predicted interacting atoms of the R28-IgI3 and CEACAM1-N binding residues.

File Name: Supplementary Data 3
Description: Bacterial strains used in this study.

File Name: Supplementary Data 4
Description: Expression vectors used in this study.
