# Supplementary material for: Human CEACAM1 is targeted by a Streptococcus pyogenes adhesin implicated in puerperal sepsis pathogenesis
Source: Nat Commun. 2023 Apr 20;14:2275. doi: 10.1038/s41467-023-37732-1 (PMC10119177; doi:10.1038/s41467-023-37732-1)
Supplement: Supplementary file 5 — Reporting Summary [file 41467_2023_37732_MOESM5_ESM.pdf]

nature portfolio

Corresponding author(s): Alex McCarthy

Last updated by author(s): 2023/03/07

Reporting Summary

Nature Portfolio wishes to improve the reproducibility of the work that we publish. This form provides structure for consistency and transparency in reporting. For further information on Nature Portfolio policies, see our Editorial Policies and the Editorial Policy Checklist.

Statistics

For all statistical analyses, confirm that the following items are present in the figure legend, table legend, main text, or Methods section.

n/a	Confirmed	
☐	☒	The exact sample size (*n*) for each experimental group/condition, given as a discrete number and unit of measurement
☐	☒	A statement on whether measurements were taken from distinct samples or whether the same sample was measured repeatedly
☐	☒	The statistical test(s) used AND whether they are one- or two-sided<br>*Only common tests should be described solely by name; describe more complex techniques in the Methods section.*
☒	☐	A description of all covariates tested
☐	☒	A description of any assumptions or corrections, such as tests of normality and adjustment for multiple comparisons
☐	☒	A full description of the statistical parameters including central tendency (e.g. means) or other basic estimates (e.g. regression coefficient) AND variation (e.g. standard deviation) or associated estimates of uncertainty (e.g. confidence intervals)
☐	☒	For null hypothesis testing, the test statistic (e.g. *F*, *t*, *r*) with confidence intervals, effect sizes, degrees of freedom and *P* value noted<br>*Give P values as exact values whenever suitable.*
☒	☐	For Bayesian analysis, information on the choice of priors and Markov chain Monte Carlo settings
☒	☐	For hierarchical and complex designs, identification of the appropriate level for tests and full reporting of outcomes
☒	☐	Estimates of effect sizes (e.g. Cohen's *d*, Pearson's *r*), indicating how they were calculated

Our web collection on statistics for biologists contains articles on many of the points above.

Software and code

Policy information about availability of computer code

Data collection	No software was used
Data analysis	Seemann T, ABRicate v0.8.13 (https://github.com/tseemann/abricate) was used to detect six regions of the spr28 gene (90-336bp in length) in bacterial genomes. Pangenome analysis and core genome alignment was performed using Panaroo and a phylogenetic tree constructed with RAxML v8 and annotated in iTOL v6. Isothermal calorimetry data was analysed in Origin 7.0. Flow cytometry data was analysed in FlowJo v10.8. Data was analysed in GraphPad Prism 8.4.3.<br><br>Crystallography data was processed and indexed with XDS, and scaled and converted to using Aimless. MOLREP was used to perform molecular replacement. Refinement was carried out by REFMAC5 and Phenix Refine. Rebuilding was carried out in Coot.

For manuscripts utilizing custom algorithms or software that are central to the research but not yet described in published literature, software must be made available to editors and reviewers. We strongly encourage code deposition in a community repository (e.g. GitHub). See the Nature Portfolio guidelines for submitting code & software for further information.

Data

Policy information about availability of data

All manuscripts must include a data availability statement. This statement should provide the following information, where applicable:

- Accession codes, unique identifiers, or web links for publicly available datasets
- A description of any restrictions on data availability
- For clinical datasets or third party data, please ensure that the statement adheres to our policy

• All data supporting the findings of this study are available within the paper or from the corresponding author upon request. Structures of R28-IgI3 in complex with CEACAM1-N (PDB ID: 8CXJ) have been deposited into the Protein Data Bank (PDB).
The manuscript also utilises data from previously published structure available on Protein Data Bank under accession numbers: 4WHD, 2GK2, 6V3P, 4QXW, 6AW2.
• This paper does not report original code.

Human research participants

Policy information about studies involving human research participants and Sex and Gender in Research.

Reporting on sex and gender	Human blood was obtained from healthy donors for studies using purified neutrophils and human whole blood. Data reported related to both sexes and all genders. Neither sex or gender were considered during design.<br><br>Human female reproductive tract sections were used for studies of CEACAM expression and infection experiments. Data reported relate to female sex. Sex was considered during design.<br><br>All samples were collected after receiving signed informed consent from all participants.
Population characteristics	All individuals were healthy. No other data was collected.
Recruitment	Donation of blood was voluntary and participants were volunteers from the Institute directly involved in the experiment. Recruitment was via word of mouth or institute emailing. Signed consent was collected. No bias on gender.<br><br>Females were recruited on a voluntary basis to donate reproductive tract specimens whom had normal cervical smears and were undergoing a planned hysterectomy for their own health. Signed consent was collected. Details are available at DOI: 10.5334/ojb.49<br><br>For immunohistochemistry, postoperative material from patients treated at the Sumy Regional Oncology Center between 2017 and 2018 (Department of Gynecology, Sumy, Ukraine) was collected. The study included 10 cases from each organ of the female reproductive system. Normal tissues were obtained from the areas of resection margins after tumor removal or from neighboring organs that were removed together during the operation. The patients were recruited by the treating physician and provided their written informed consent for tissue investigation. The written informed consent remained in the patient's file (Inpatient Health Record).
Ethics oversight	Human blood was obtained from healthy donors, approved by the Regional Ethics Committee and Imperial College Healthcare NHS Trust Tissue Bank (Regional Ethics Committee approval no. 17/WA/0161, Imperial College Healthcare Tissue Bank Human Tissue Authority license no. 12275, and Imperial College Research Ethics Committee no. 19IC5166).<br>Human female reproductive tract organ specimens (tissues of vagina, ectocervix, endocervix, uterus, Fallopian tube, and ovary) for immunohistochemistry were obtained from patients treated at the Department of Gynecology of Sumy Regional Oncology Center, Sumy, Ukraine. All tissues were collected after receiving signed informed consent from all patients. The Bioethics Commission of the Medical Institute of Sumy State University approved the experimental protocol (no. 36 from 14.05.2018). Surgically resected specimens of human ecto-cervical tissue were obtained from the York Tissue Bank, University of York, UK approved by the Yorkshire & The Humber-Leeds East Research Ethics Committee (NHS REC 20/YH/0126) or collected at St. Mary’s Hospital, Imperial College Healthcare NHS Trust, London, UK, after receiving signed informed consent from all patients through the Imperial College Healthcare Tissue Bank approved by Research Ethics Committee (IRAS 17/WA/0161).

Note that full information on the approval of the study protocol must also be provided in the manuscript.

Field-specific reporting

Please select the one below that is the best fit for your research. If you are not sure, read the appropriate sections before making your selection.

☒ Life sciences ☐ Behavioural & social sciences ☐ Ecological, evolutionary & environmental sciences

For a reference copy of the document with all sections, see nature.com/documents/nr-reporting-summary-flat.pdf

Life sciences study design

All studies must disclose on these points even when the disclosure is negative.

Sample size	Sample sizes were not predetermined based on statistical methods, but were chosen according to the standards in the field - at least 3 independent biological replicates. This typically generated sufficient data for statistical analysis.
Data exclusions	No data were excluded from analysis
Replication	Numbers of experimental replicates are stated in each figure legend. Reported results were consistently replicated across multiple experiments with all replicates generating similar results.
Randomization	No randomization was necessary as experiments were performed with appropriate controls. Randomization is not generally used in this field
Blinding	Investigators were not blinded. Blinding during analysis was not necessary because the results are quantitative and did not require subjective judgment or interpretation. Blinding is not typically used in the field.

Reporting for specific materials, systems and methods

We require information from authors about some types of materials, experimental systems and methods used in many studies. Here, indicate whether each material, system or method listed is relevant to your study. If you are not sure if a list item applies to your research, read the appropriate section before selecting a response.

Materials & experimental systems

n/a	Involved in the study	
☐	☒	Antibodies
☐	☒	Eukaryotic cell lines
☒	☐	Palaeontology and archaeology
☒	☐	Animals and other organisms
☒	☐	Clinical data
☒	☐	Dual use research of concern

Methods

n/a	Involved in the study	
☒	☐	ChIP-seq
☐	☒	Flow cytometry
☒	☐	MRI-based neuroimaging

Antibodies

Antibodies used	Mouse monoclocal antibodies for CEACAMs provided by author BB Singer at LeukoCom B3-17 (MABT397), 5C8C4 (153325), C5-1X/8, 3E10-3 (153322), 1H7-4B (153324) at concentrations of 5 ug/mL (expression) or 20 ug/mL (inhibition). mAb CC1/3/5-Sab provided by BB Singer.<br><br>Rabbit polyclonal antibodies for detecting CEACAMs supplied by LeukoCom (154003) at (1:400 ELISA) or ()<br>Anti-His-FITC supplied from Thermofisher (MA181891) at 1:50 dilution.<br>Goat anti-mouse-IgG-PE from Life Technologies (12-4010-87) at 1:1,000 dilution<br>Goat anti-rabbit-IgG-HRP from Life Technologies (A16096) at 1:10,000 dilution<br>Goat anti-mouse-IgG-HRP from Life Technologies (A16066) at 1:10,000 dilution<br>Goat anti-rabbit-IgG-AF647 from Invitrogen (A-21245) at 1:500 dilution.<br>Mouse IgG1 isotype from R&D biosystems (MAB002) at concentrations of 5 ug/mL (expression) or 20 ug/mL (inhibition).
Validation	Validation statements on manufacturers websites. Specificity of primary antibodies was tested against recombinant proteins in ELISA or Western blot or flow cytometry assays. Detection with secondary antibodies validated by ELISA, Western Blot or flow cytometry assays.

Eukaryotic cell lines

Policy information about cell lines and Sex and Gender in Research

Cell line source(s)	ME-180 (female human cervical cell line) cells received from AMS Biotechnology. EXPI293f cells received from Thermo Fisher Scientific. HEK cell lines generated by Retrogenix, UK. HeLa (female human cervical cell line) cell lines received from Jos van Putten (Utrecht, the Netherlands), previously published in PMID: 9689124. Original Source unknown. CHO cells previously published PMID: 32064046. Original source unknown. CHO cells lines constructed by BB Singer.
Authentication	No authentication.
Mycoplasma contamination	HeLa cell lines tested negative for mycoplasma contamination. CHO and ME-180 cells not tested for mycoplasma contamination.

Commonly misidentified lines (See ICLAC register)	no commonly misidentified lines were used in this study

Flow Cytometry

Plots

Confirm that:

☒ The axis labels state the marker and fluorochrome used (e.g. CD4-FITC).

☒ The axis scales are clearly visible. Include numbers along axes only for bottom left plot of group (a 'group' is an analysis of identical markers).

☒ All plots are contour plots with outliers or pseudocolor plots.

☒ A numerical value for number of cells or percentage (with statistics) is provided.

Methodology

Sample preparation	Bacterial cells prepared by culture in appropriate media. ME-180 cells cultured as detailed in methods. Neutrophils purified as detailed in methods.
Instrument	Data collected on BD FACSCalibur™ Flow Cytometer - BD Biosciences, or Amnis CellStream® Benchtop Flow Cytometer from Luminex
Software	Data collected BD FACStation™ software or CellStream™ Acquisition and Analysis Software<br>Data analysed FlowJo v10.8.0.
Cell population abundance	Cells were not sorted.
Gating strategy	Bacteria populations gated on FSC/SSC.<br>Neutrophils gated on FSC/SSC.<br>ME-180 cells gated on FSC/SSC.

☒ Tick this box to confirm that a figure exemplifying the gating strategy is provided in the Supplementary Information.
